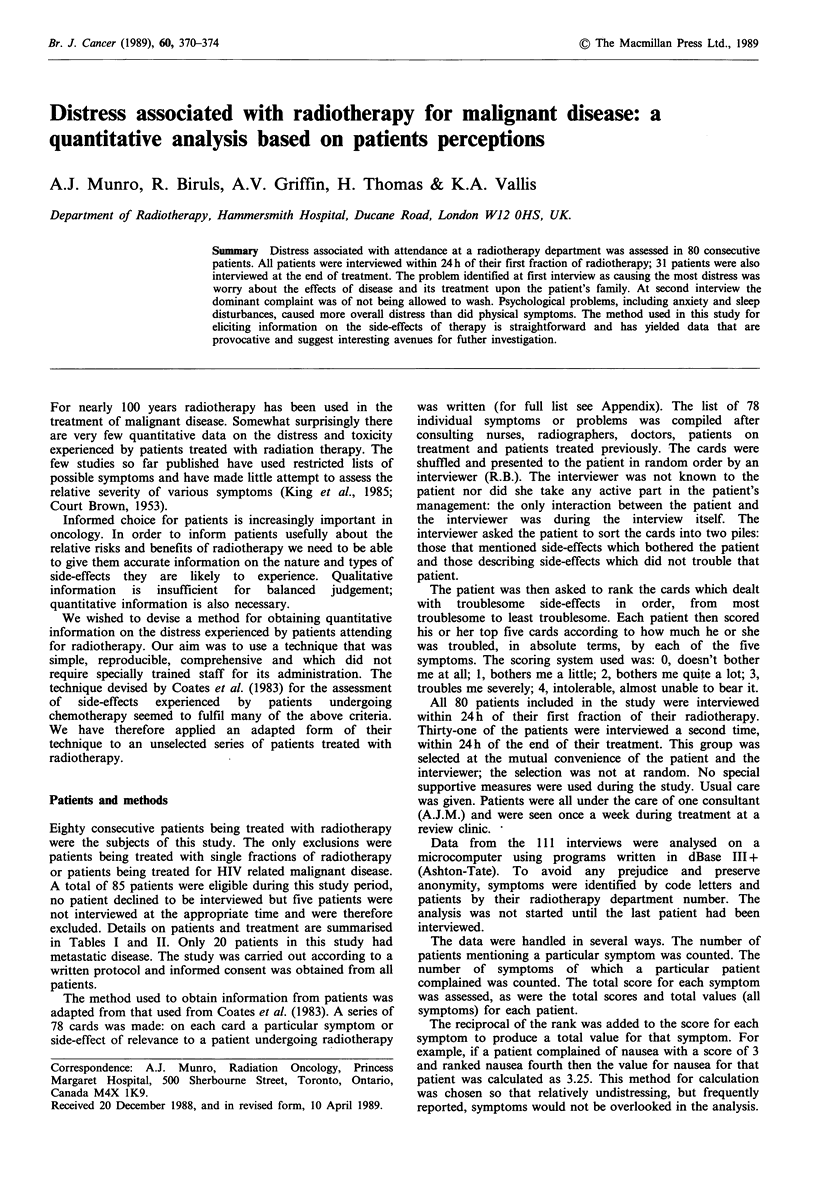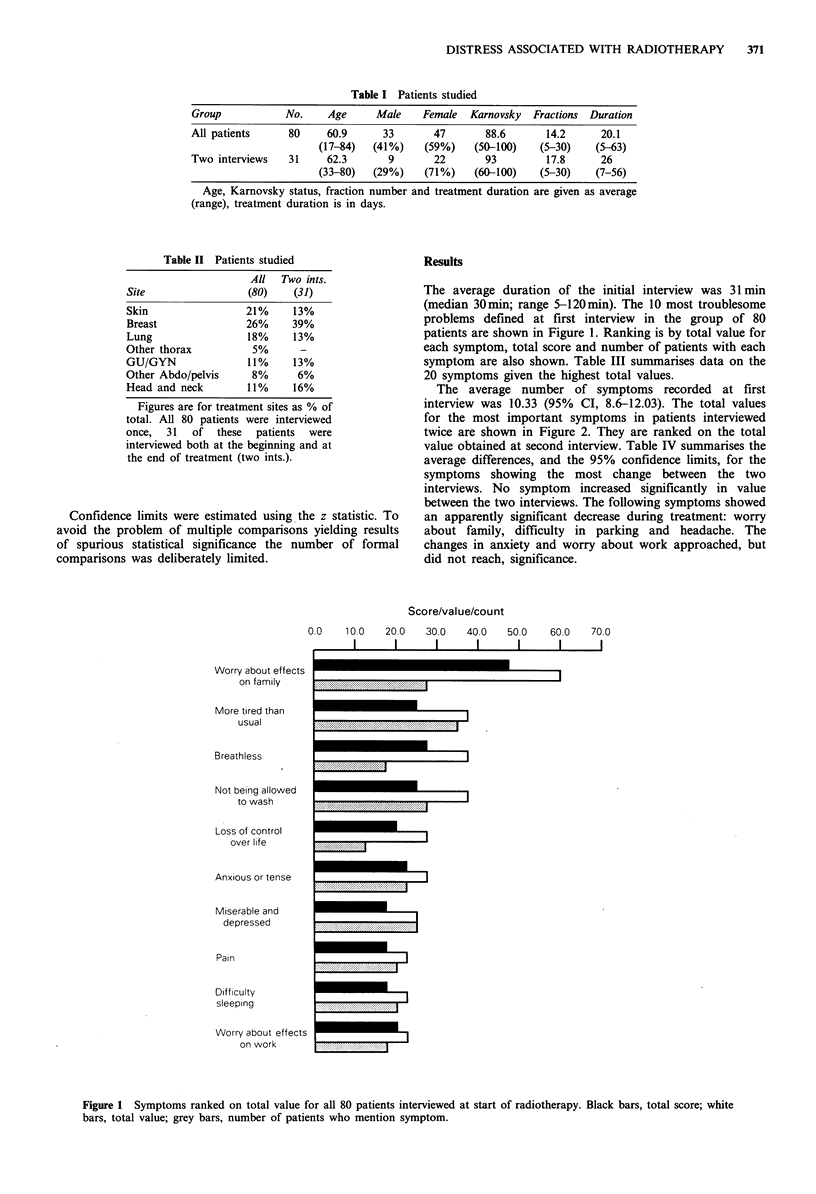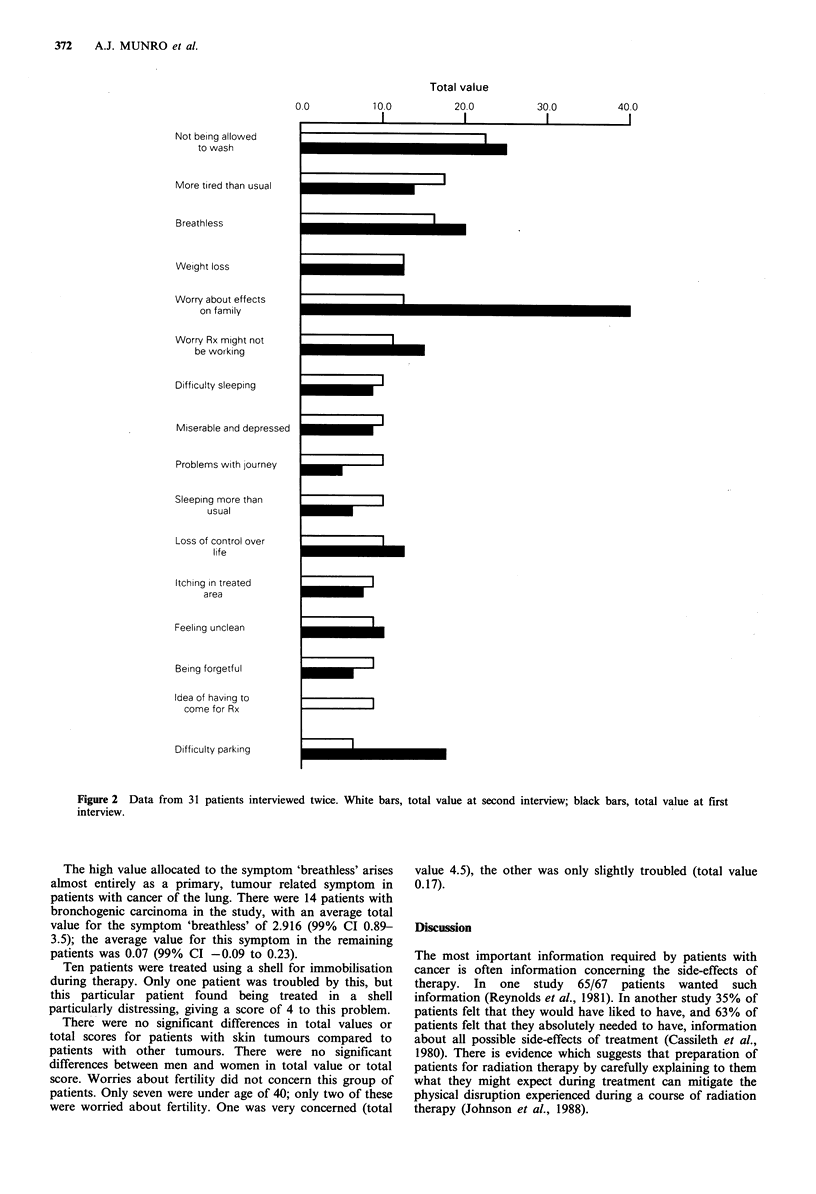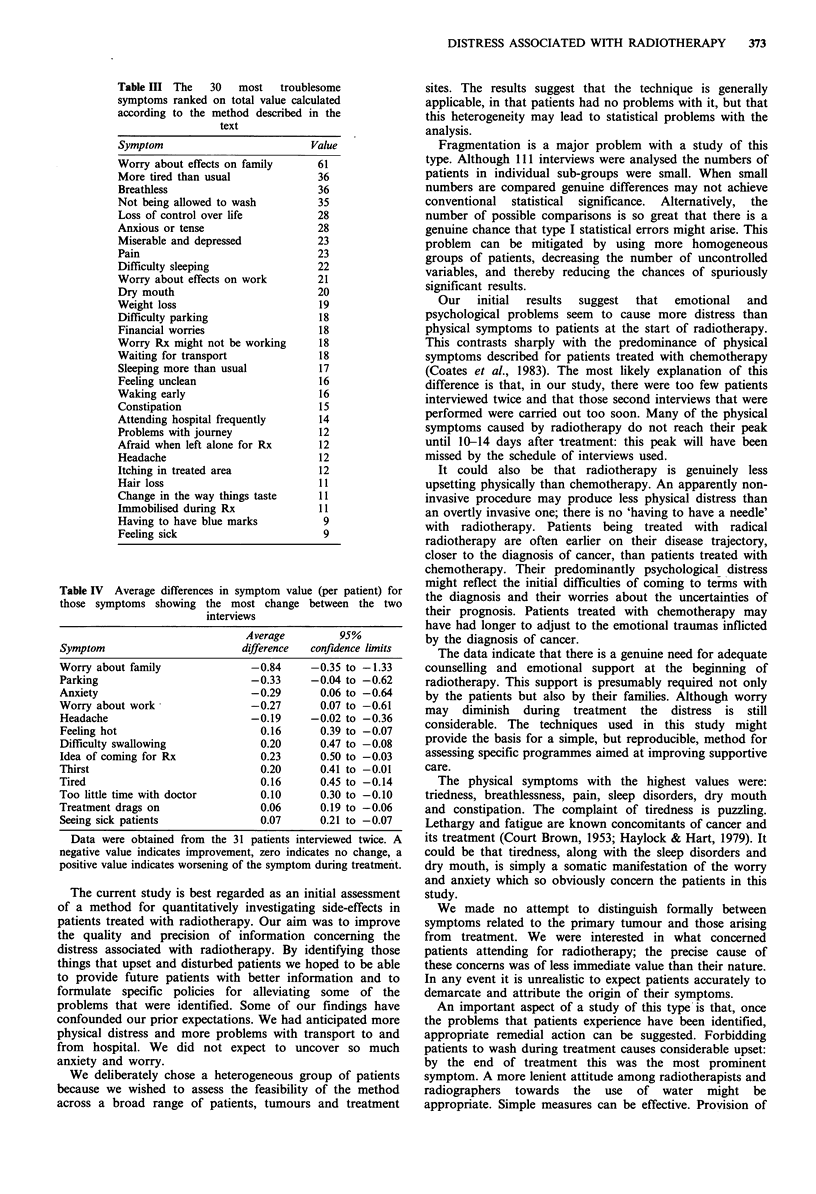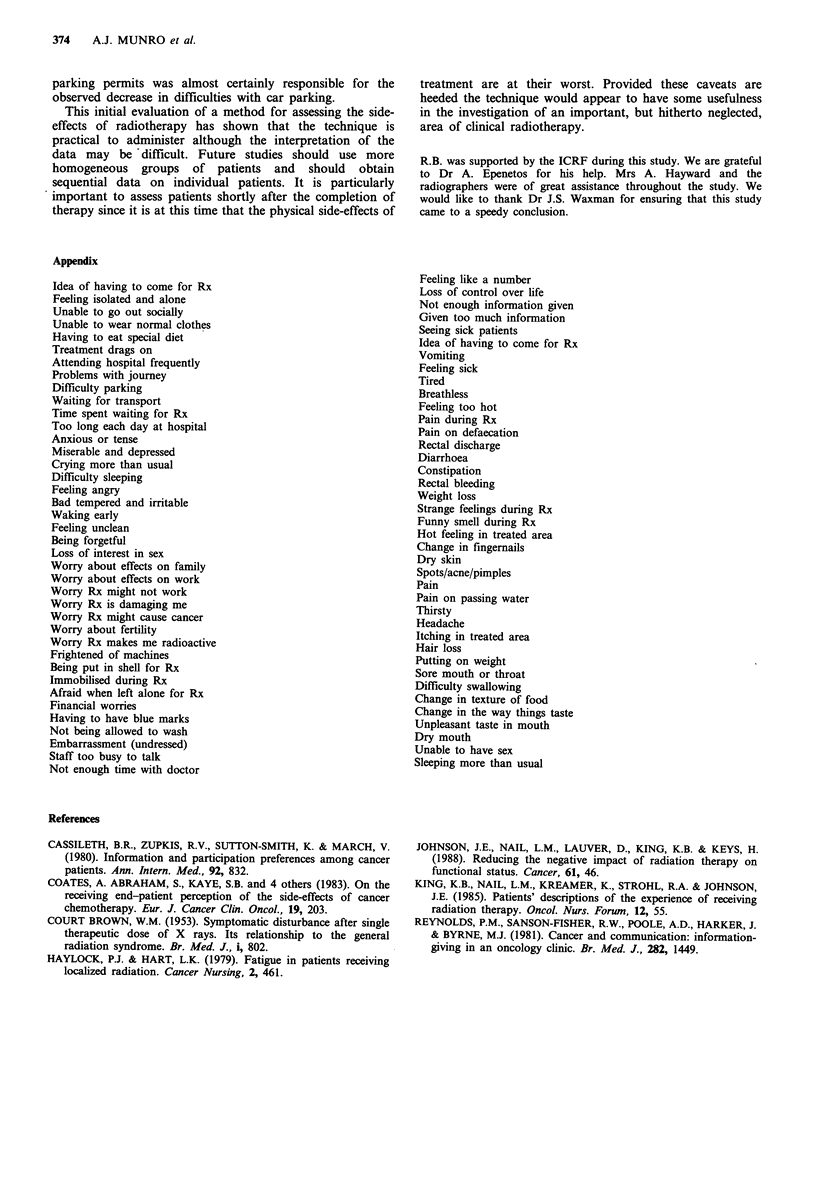# Distress associated with radiotherapy for malignant disease: a quantitative analysis based on patients perceptions.

**DOI:** 10.1038/bjc.1989.287

**Published:** 1989-09

**Authors:** A. J. Munro, R. Biruls, A. V. Griffin, H. Thomas, K. A. Vallis

**Affiliations:** Department of Radiotherapy, Hammersmith Hospital, London, UK.

## Abstract

Distress associated with attendance at a radiotherapy department was assessed in 80 consecutive patients. All patients were interviewed within 24 h of their first fraction of radiotherapy; 31 patients were also interviewed at the end of treatment. The problem identified at first interview as causing the most distress was worry about the effects of disease and its treatment upon the patient's family. At second interview the dominant complaint was of not being allowed to wash. Psychological problems, including anxiety and sleep disturbances, caused more overall distress than did physical symptoms. The method used in this study for eliciting information on the side-effects of therapy is straightforward and has yielded data that are provocative and suggest interesting avenues for further investigation.


*Br. J. Cancer* (1989), **60**, 370–374 © The Macmillan Press Ltd., 1989

### Distress associated with radiotherapy for malignant disease: a quantitative analysis based on patients perceptions

A.J. Munro, R. Biruls, A.V. Griffin, H. Thomas & K.A. Vallis

*Department of Radiotherapy, Hammersmith Hospital, Ducane Road, London W12 0HS, UK.*

**Summary** Distress associated with attendance at a radiotherapy department was assessed in 80 consecutive patients. All patients were interviewed within 24 h of their first fraction of radiotherapy; 31 patients were also interviewed at the end of treatment. The problem identified at first interview as causing the most distress was worry about the effects of disease and its treatment upon the patient's family. At second interview the dominant complaint was of not being allowed to wash. Psychological problems, including anxiety and sleep disturbances, caused more overall distress than did physical symptoms. The method used in this study for eliciting information on the side-effects of therapy is straightforward and has yielded data that are provocative and suggest interesting avenues for futher investigation.

For nearly 100 years radiotherapy has been used in the treatment of malignant disease. Somewhat surprisingly there are very few quantitative data on the distress and toxicity experienced by patients treated with radiation therapy. The few studies so far published have used restricted lists of possible symptoms and have made little attempt to assess the relative severity of various symptoms (King *et al.*, 1985; Court Brown, 1953).

Informed choice for patients is increasingly important in oncology. In order to inform patients usefully about the relative risks and benefits of radiotherapy we need to be able to give them accurate information on the nature and types of side-effects they are likely to experience. Qualitative information is insufficient for balanced judgement; quantitative information is also necessary.

We wished to devise a method for obtaining quantitative information on the distress experienced by patients attending for radiotherapy. Our aim was to use a technique that was simple, reproducible, comprehensive and which did not require specially trained staff for its administration. The technique devised by Coates *et al.* (1983) for the assessment of side-effects experienced by patients undergoing chemotherapy seemed to fulfil many of the above criteria. We have therefore applied an adapted form of their technique to an unselected series of patients treated with radiotherapy.

### Patients and methods

Eighty consecutive patients being treated with radiotherapy were the subjects of this study. The only exclusions were patients being treated with single fractions of radiotherapy or patients being treated for HIV related malignant disease. A total of 85 patients were eligible during this study period, no patient declined to be interviewed but five patients were not interviewed at the appropriate time and were therefore excluded. Details on patients and treatment are summarised in Tables I and II. Only 20 patients in this study had metastatic disease. The study was carried out according to a written protocol and informed consent was obtained from all patients.

The method used to obtain information from patients was adapted from that used from Coates *et al.* (1983). A series of 78 cards was made: on each card a particular symptom or side-effect of relevance to a patient undergoing radiotherapy was written (for full list see Appendix). The list of 78 individual symptoms or problems was compiled after consulting nurses, radiographers, doctors, patients on treatment and patients treated previously. The cards were shuffled and presented to the patient in random order by an interviewer (R.B.). The interviewer was not known to the patient nor did she take any active part in the patient's management: the only interaction between the patient and the interviewer was during the interview itself. The interviewer asked the patient to sort the cards into two piles: those that mentioned side-effects which bothered the patient and those describing side-effects which did not trouble that patient.

The patient was then asked to rank the cards which dealt with troublesome side-effects in order, from most troublesome to least troublesome. Each patient then scored his or her top five cards according to how much he or she was troubled, in absolute terms, by each of the five symptoms. The scoring system used was: 0, doesn't bother me at all; 1, bothers me a little; 2, bothers me quite a lot; 3, troubles me severely; 4, intolerable, almost unable to bear it.

All 80 patients included in the study were interviewed within 24 h of their first fraction of their radiotherapy. Thirty-one of the patients were interviewed a second time, within 24 h of the end of their treatment. This group was selected at the mutual convenience of the patient and the interviewer; the selection was not at random. No special supportive measures were used during the study. Usual care was given. Patients were all under the care of one consultant (A.J.M.) and were seen once a week during treatment at a review clinic.

Data from the 111 interviews were analysed on a microcomputer using programs written in dBase III+ (Ashton-Tate). To avoid any prejudice and preserve anonymity, symptoms were identified by code letters and patients by their radiotherapy department number. The analysis was not started until the last patient had been interviewed.

The data were handled in several ways. The number of patients mentioning a particular symptom was counted. The number of symptoms of which a particular patient complained was counted. The total score for each symptom was assessed, as were the total scores and total values (all symptoms) for each patient.

The reciprocal of the rank was added to the score for each symptom to produce a total value for that symptom. For example, if a patient complained of nausea with a score of 3 and ranked nausea fourth then the value for nausea for that patient was calculated as 3.25. This method for calculation was chosen so that relatively undistressing, but frequently reported, symptoms would not be overlooked in the analysis.

Correspondence: A.J. Munro, Radiation Oncology, Princess Margaret Hospital, 500 Sherbourne Street, Toronto, Ontario, Canada M4X 1K9.
Received 20 December 1988, and in revised form, 10 April 1989.

**Table I** Patients studied

| *Group* | *No.* | *Age* | *Male* | *Female* | *Karnovsky* | *Fractions* | *Duration* |
|---|---|---|---|---|---|---|---|
| All patients | 80 | 60.9 (17–84) | 33 (41%) | 47 (59%) | 88.6 (50–100) | 14.2 (5–30) | 20.1 (5–63) |
| Two interviews | 31 | 62.3 (33–80) | 9 (29%) | 22 (71%) | 93 (60–100) | 17.8 (5–30) | 26 (7–56) |

Age, Karnovsky status, fraction number and treatment duration are given as average (range), treatment duration is in days.

**Table II** Patients studied

| *Site* | *All* (*80*) | *Two ints.* (*31*) |
|---|---|---|
| Skin | 21% | 13% |
| Breast | 26% | 39% |
| Lung | 18% | 13% |
| Other thorax | 5% | – |
| GU/GYN | 11% | 13% |
| Other Abdo/pelvis | 8% | 6% |
| Head and neck | 11% | 16% |

Figures are for treatment sites as % of total. All 80 patients were interviewed once, 31 of these patients were interviewed both at the beginning and at the end of treatment (two ints.).

Confidence limits were estimated using the $z$ statistic. To avoid the problem of multiple comparisons yielding results of spurious statistical significance the number of formal comparisons was deliberately limited.

### Results

The average duration of the initial interview was 31 min (median 30 min; range 5–120 min). The 10 most troublesome problems defined at first interview in the group of 80 patients are shown in Figure 1. Ranking is by total value for each symptom, total score and number of patients with each symptom are also shown. Table III summarises data on the 20 symptoms given the highest total values.

The average number of symptoms recorded at first interview was 10.33 (95% CI, 8.6–12.03). The total values for the most important symptoms in patients interviewed twice are shown in Figure 2. They are ranked on the total value obtained at second interview. Table IV summarises the average differences, and the 95% confidence limits, for the symptoms showing the most change between the two interviews. No symptom increased significantly in value between the two interviews. The following symptoms showed an apparently significant decrease during treatment: worry about family, difficulty in parking and headache. The changes in anxiety and worry about work approached, but did not reach, significance.

**Figure 1** Symptoms ranked on total value for all 80 patients interviewed at start of radiotherapy. Black bars, total score; white bars, total value; grey bars, number of patients who mention symptom.

**Figure 2** Data from 31 patients interviewed twice. White bars, total value at second interview; black bars, total value at first interview.

The high value allocated to the symptom 'breathless' arises almost entirely as a primary, tumour related symptom in patients with cancer of the lung. There were 14 patients with bronchogenic carcinoma in the study, with an average total value for the symptom 'breathless' of 2.916 (99% CI 0.89–3.5); the average value for this symptom in the remaining patients was 0.07 (99% CI −0.09 to 0.23).

Ten patients were treated using a shell for immobilisation during therapy. Only one patient was troubled by this, but this particular patient found being treated in a shell particularly distressing, giving a score of 4 to this problem.

There were no significant differences in total values or total scores for patients with skin tumours compared to patients with other tumours. There were no significant differences between men and women in total value or total score. Worries about fertility did not concern this group of patients. Only seven were under age of 40; only two of these were worried about fertility. One was very concerned (total value 4.5), the other was only slightly troubled (total value 0.17).

### Discussion

The most important information required by patients with cancer is often information concerning the side-effects of therapy. In one study 65/67 patients wanted such information (Reynolds *et al.*, 1981). In another study 35% of patients felt that they would have liked to have, and 63% of patients felt that they absolutely needed to have, information about all possible side-effects of treatment (Cassileth *et al.*, 1980). There is evidence which suggests that preparation of patients for radiation therapy by carefully explaining to them what they might expect during treatment can mitigate the physical disruption experienced during a course of radiation therapy (Johnson *et al.*, 1988).

**Table III** The 30 most troublesome symptoms ranked on total value calculated according to the method described in the text

| *Symptom* | *Value* |
|---|---|
| Worry about effects on family | 61 |
| More tired than usual | 36 |
| Breathless | 36 |
| Not being allowed to wash | 35 |
| Loss of control over life | 28 |
| Anxious or tense | 28 |
| Miserable and depressed | 23 |
| Pain | 23 |
| Difficulty sleeping | 22 |
| Worry about effects on work | 21 |
| Dry mouth | 20 |
| Weight loss | 19 |
| Difficulty parking | 18 |
| Financial worries | 18 |
| Worry Rx might not be working | 18 |
| Waiting for transport | 18 |
| Sleeping more than usual | 17 |
| Feeling unclean | 16 |
| Waking early | 16 |
| Constipation | 15 |
| Attending hospital frequently | 14 |
| Problems with journey | 12 |
| Afraid when left alone for Rx | 12 |
| Headache | 12 |
| Itching in treated area | 12 |
| Hair loss | 11 |
| Change in the way things taste | 11 |
| Immobilised during Rx | 11 |
| Having to have blue marks | 9 |
| Feeling sick | 9 |

**Table IV** Average differences in symptom value (per patient) for those symptoms showing the most change between the two interviews

| *Symptom* | *Average difference* | *95% confidence limits* |
|---|---|---|
| Worry about family | −0.84 | −0.35 to −1.33 |
| Parking | −0.33 | −0.04 to −0.62 |
| Anxiety | −0.29 | 0.06 to −0.64 |
| Worry about work | −0.27 | 0.07 to −0.61 |
| Headache | −0.19 | −0.02 to −0.36 |
| Feeling hot | 0.16 | 0.39 to −0.07 |
| Difficulty swallowing | 0.20 | 0.47 to −0.08 |
| Idea of coming for Rx | 0.23 | 0.50 to −0.03 |
| Thirst | 0.20 | 0.41 to −0.01 |
| Tired | 0.16 | 0.45 to −0.14 |
| Too little time with doctor | 0.10 | 0.30 to −0.10 |
| Treatment drags on | 0.06 | 0.19 to −0.06 |
| Seeing sick patients | 0.07 | 0.21 to −0.07 |

Data were obtained from the 31 patients interviewed twice. A negative value indicates improvement, zero indicates no change, a positive value indicates worsening of the symptom during treatment.

The current study is best regarded as an initial assessment of a method for quantitatively investigating side-effects in patients treated with radiotherapy. Our aim was to improve the quality and precision of information concerning the distress associated with radiotherapy. By identifying those things that upset and disturbed patients we hoped to be able to provide future patients with better information and to formulate specific policies for alleviating some of the problems that were identified. Some of our findings have confounded our prior expectations. We had anticipated more physical distress and more problems with transport to and from hospital. We did not expect to uncover so much anxiety and worry.

We deliberately chose a heterogeneous group of patients because we wished to assess the feasibility of the method across a broad range of patients, tumours and treatment sites. The results suggest that the technique is generally applicable, in that patients had no problems with it, but that this heterogeneity may lead to statistical problems with the analysis.

Fragmentation is a major problem with a study of this type. Although 111 interviews were analysed the numbers of patients in individual sub-groups were small. When small numbers are compared genuine differences may not achieve conventional statistical significance. Alternatively, the number of possible comparisons is so great that there is a genuine chance that type I statistical errors might arise. This problem can be mitigated by using more homogeneous groups of patients, decreasing the number of uncontrolled variables, and thereby reducing the chances of spuriously significant results.

Our initial results suggest that emotional and psychological problems seem to cause more distress than physical symptoms to patients at the start of radiotherapy. This contrasts sharply with the predominance of physical symptoms described for patients treated with chemotherapy (Coates *et al.*, 1983). The most likely explanation of this difference is that, in our study, there were too few patients interviewed twice and that those second interviews that were performed were carried out too soon. Many of the physical symptoms caused by radiotherapy do not reach their peak until 10–14 days after treatment: this peak will have been missed by the schedule of interviews used.

It could also be that radiotherapy is genuinely less upsetting physically than chemotherapy. An apparently non-invasive procedure may produce less physical distress than an overtly invasive one; there is no 'having to have a needle' with radiotherapy. Patients being treated with radical radiotherapy are often earlier on their disease trajectory, closer to the diagnosis of cancer, than patients treated with chemotherapy. Their predominantly psychological distress might reflect the initial difficulties of coming to terms with the diagnosis and their worries about the uncertainties of their prognosis. Patients treated with chemotherapy may have had longer to adjust to the emotional traumas inflicted by the diagnosis of cancer.

The data indicate that there is a genuine need for adequate counselling and emotional support at the beginning of radiotherapy. This support is presumably required not only by the patients but also by their families. Although worry may diminish during treatment the distress is still considerable. The techniques used in this study might provide the basis for a simple, but reproducible, method for assessing specific programmes aimed at improving supportive care.

The physical symptoms with the highest values were: triedness, breathlessness, pain, sleep disorders, dry mouth and constipation. The complaint of tiredness is puzzling. Lethargy and fatigue are known concomitants of cancer and its treatment (Court Brown, 1953; Haylock & Hart, 1979). It could be that tiredness, along with the sleep disorders and dry mouth, is simply a somatic manifestation of the worry and anxiety which so obviously concern the patients in this study.

We made no attempt to distinguish formally between symptoms related to the primary tumour and those arising from treatment. We were interested in what concerned patients attending for radiotherapy; the precise cause of these concerns was of less immediate value than their nature. In any event it is unrealistic to expect patients accurately to demarcate and attribute the origin of their symptoms.

An important aspect of a study of this type is that, once the problems that patients experience have been identified, appropriate remedial action can be suggested. Forbidding patients to wash during treatment causes considerable upset: by the end of treatment this was the most prominent symptom. A more lenient attitude among radiotherapists and radiographers towards the use of water might be appropriate. Simple measures can be effective. Provision of parking permits was almost certainly responsible for the observed decrease in difficulties with car parking.

This initial evaluation of a method for assessing the side-effects of radiotherapy has shown that the technique is practical to administer although the interpretation of the data may be difficult. Future studies should use more homogeneous groups of patients and should obtain sequential data on individual patients. It is particularly important to assess patients shortly after the completion of therapy since it is at this time that the physical side-effects of treatment are at their worst. Provided these caveats are heeded the technique would appear to have some usefulness in the investigation of an important, but hitherto neglected, area of clinical radiotherapy.

R.B. was supported by the ICRF during this study. We are grateful to Dr A. Epenetos for his help. Mrs A. Hayward and the radiographers were of great assistance throughout the study. We would like to thank Dr J.S. Waxman for ensuring that this study came to a speedy conclusion.

**Appendix**

Idea of having to come for Rx
Feeling isolated and alone
Unable to go out socially
Unable to wear normal clothes
Having to eat special diet
Treatment drags on
Attending hospital frequently
Problems with journey
Difficulty parking
Waiting for transport
Time spent waiting for Rx
Too long each day at hospital
Anxious or tense
Miserable and depressed
Crying more than usual
Difficulty sleeping
Feeling angry
Bad tempered and irritable
Waking early
Feeling unclean
Being forgetful
Loss of interest in sex
Worry about effects on family
Worry about effects on work
Worry Rx might not work
Worry Rx is damaging me
Worry Rx might cause cancer
Worry about fertility
Worry Rx makes me radioactive
Frightened of machines
Being put in shell for Rx
Immobilised during Rx
Afraid when left alone for Rx
Financial worries
Having to have blue marks
Not being allowed to wash
Embarrassment (undressed)
Staff too busy to talk
Not enough time with doctor
Feeling like a number
Loss of control over life
Not enough information given
Given too much information
Seeing sick patients
Idea of having to come for Rx
Vomiting
Feeling sick
Tired
Breathless
Feeling too hot
Pain during Rx
Pain on defaecation
Rectal discharge
Diarrhoea
Constipation
Rectal bleeding
Weight loss
Strange feelings during Rx
Funny smell during Rx
Hot feeling in treated area
Change in fingernails
Dry skin
Spots/acne/pimples
Pain
Pain on passing water
Thirsty
Headache
Itching in treated area
Hair loss
Putting on weight
Sore mouth or throat
Difficulty swallowing
Change in texture of food
Change in the way things taste
Unpleasant taste in mouth
Dry mouth
Unable to have sex
Sleeping more than usual

**References**

CASSILETH, B.R., ZUPKIS, R.V., SUTTON-SMITH, K. & MARCH, V. (1980). Information and participation preferences among cancer patients. *Ann. Intern. Med.*, **92**, 832.

COATES, A. ABRAHAM, S., KAYE, S.B. and 4 others (1983). On the receiving end–patient perception of the side-effects of cancer chemotherapy. *Eur. J. Cancer Clin. Oncol.*, **19**, 203.

COURT BROWN, W.M. (1953). Symptomatic disturbance after single therapeutic dose of X rays. Its relationship to the general radiation syndrome. *Br. Med. J.*, **i**, 802.

HAYLOCK, P.J. & HART, L.K. (1979). Fatigue in patients receiving localized radiation. *Cancer Nursing*, **2**, 461.

JOHNSON, J.E., NAIL, L.M., LAUVER, D., KING, K.B. & KEYS, H. (1988). Reducing the negative impact of radiation therapy on functional status. *Cancer*, **61**, 46.

KING, K.B., NAIL, L.M., KREAMER, K., STROHL, R.A. & JOHNSON, J.E. (1985). Patients' descriptions of the experience of receiving radiation therapy. *Oncol. Nurs. Forum*, **12**, 55.

REYNOLDS, P.M., SANSON-FISHER, R.W., POOLE, A.D., HARKER, J. & BYRNE, M.J. (1981). Cancer and communication: information-giving in an oncology clinic. *Br. Med. J.*, **282**, 1449.